# Cardiovascular and Cerebral Responses During a Vasovagal Reaction Without Syncope

**DOI:** 10.3389/fnins.2019.01315

**Published:** 2019-12-10

**Authors:** Mathias R. Aebi, Nicolas Bourdillon, Hadj B. Meziane, Edward Nicol, Jérôme Barral, Grégoire P. Millet, Denis Bron

**Affiliations:** ^1^Institute of Sport Sciences, University of Lausanne, Lausanne, Switzerland; ^2^Aeromedical Center (AeMC), Swiss Air Force, Dübendorf, Switzerland; ^3^Institute of Psychology, Faculty of Social and Political Sciences, University of Lausanne, Lausanne, Switzerland; ^4^Aviation Medicine Clinical Service, RAF Centre of Aviation Medicine, RAF Henlow, Bedfordshire, United Kingdom

**Keywords:** vasovagal mechanism, pre-syncope symptoms, hypotension and bradycardia, cerebral hypoperfusion, EEG flattening and slowing

## Abstract

This clinical case report presents synchronous physiological data from an individual in whom a spontaneous vasovagal reaction occurred without syncope. The physiological data are presented for three main phases: Baseline (0–200 s), vasovagal reaction (200–600 s), and recovery period (600–1200 s). The first physiological changes occurred at around 200 s, with a decrease in blood pressure, peak in heart rate and vastus lateralis tissue oxygenation, and a drop in alpha power. The vasovagal reaction was associated with a progressive decrease in blood pressure, heart rate and cerebral oxygenation, whilst the mean middle cerebral artery blood flow velocity and blood oxygen saturation remained unchanged. Heart rate variability parameters indicated significant parasympathetic activation with a decrease in sympathetic tone and increased baroreflex sensitivity. The total blood volume and tissue oxygenation index (TOI) dropped in the brain but slightly increased in the vastus lateralis, suggesting cerebral hypoperfusion with blood volume pooling in the lower body part. Cerebral hypoperfusion during the vasovagal reaction was associated with electroencephalography (EEG) flattening (i.e., decreased power in beta and theta activity) followed by an EEG high-amplitude “slow” phase (i.e., increased power in theta activity). The subject developed signs and symptoms of pre-syncope with EEG flattening and slowing during prolonged periods of symptomatic hypotension, but did not lose consciousness.

## Introduction

Vasovagal reactions include arterial vasodilation and bradycardia as mechanisms that may precipitate a syncopal response (Lewis, 1932); defined as a transient loss of consciousness caused by cerebral hypoperfusion followed by spontaneous recovery (Freeman et al., 2011). Syncope is a common cause of transient loss of consciousness among children and adults (Ganzeboom et al., 2003). Vaso-vagal events are usually triggered by parasympathetic over-activation associated with a reduced sympathetic response (Gunnar Wallin and Sundlöf, 1982), which in turn causes a reduction in cardiac output, hypotension and cerebral hypoperfusion (Wieling et al., 2016). Arterial blood pressure decreases below the auto-regulatory threshold (Van Lieshout et al., 2003) and cannot be compensated for by the delayed normal auto-regulatory reflex-loop blood pressure adjustment of the baroreflex (Chapleau, 2003), leading to cerebral hypoperfusion (Lund et al., 2017). Furthermore, parasympathetic activation corresponds to an increase in heart rate variability and is related with slow alpha power on the electroencephalography (EEG), mainly in the frontal area (Takahashi et al., 2005). Individuals with anxiety often exhibit a desynchronization in alpha frequency during attentional tasks (Ward et al., 2018). The EEG signal shows EEG slowing during vasovagal reaction without and with syncope (loss of consciousness). Hypotension without syncope is associated with EEG slowing (Heyer et al., 2016) and may be associated with prolonged hypotension (Heyer, 2019). EEG slowing corresponds with a shift from high to low frequencies. Previous reports on vasovagal syncope describe two types of central nervous system activity: a reduction in brain-wave amplitude during hypotension (Ammirati et al., 1998), with EEG signal flattening during cerebral hypoperfusion (Dijk et al., 2014). The usual symptoms of vasovagal reactions are warmth, nausea, altered concentration and visual disturbance (Jardine et al., 2018). Despite the numerous mechanisms involved, there is a scarcity of synchronous physiological data measured during spontaneous vasovagal reactions. This case study reports continuous physiological changes with simultaneous EEG results during a spontaneous and unexpected vasovagal reaction without loss of consciousness, which occurred in a young individual participating in a clinical trial.

## Participant Characteristics

The 20-year-old subject was a tall, thin (178 cm, 52.3 kg, BMI 16.5) male flight attendant with no significant medical history. The subject was participating in a study investigating cerebral responses to low intensity cycling, in a small room of 22 m^3^ in which he may have felt confined. No other participants (*n* = 20) reported discomfort during the clinical trial. The initial electrocardiogram (performed supine and at rest before study enrolment) was normal (PR: 110 ms, PQ: 140 ms, QRS: 96 ms, QT: 386 ms and QTc: 410 ms, no ST/T changes). The vasovagal reaction occurred unexpectedly at rest directly after the start of recordings. The subject remained conscious during vasovagal reaction but showed decreased postural tone and auditory impairment. The study he was participating in was approved by the ethical committee of Zürich, Switzerland (2018-00006). Written informed consent was obtained before study enrolment and for the publication of this case report. This clinical trial is accessible on ClinicalTrials.gov (NCT03439202).

## Materials and Methods

Electroencephalography activity signals were recorded from 19 bipolar EEG channels and sampled at 200 Hz. Pre-processing was carried out with custom-written MATLAB code (MathWorks Inc.) and the EEGLAB analysis tools (Delorme and Makeig, 2004). The data set was filtered between 0.5 and 70 Hz using a zero-phase Butterworth filter (Notch-filter was set to 50 Hz) before an independent component analysis was used to remove blink artifacts. The signals were re-referenced to a common average. A time-frequency analysis based on a continuous complex Morlet’s wavelet transformation of the signal (between 1 and 45 Hz with 0.5-Hz steps) was carried out. Time-frequency power values were converted to decibel units (dB) and a baseline was calculated as the mean value of the first 60 s of the signal subtracted from the time-frequency power of the whole data. Power values in theta (4–7 Hz), alpha (8–13 Hz), beta (13.5–30 Hz), and gamma (30.5–45 Hz) frequency bands were averaged in periods of 1 min for each electrode.

Continuous blood pressure was measured at the middle and index finger of the left hand using a double pneumatic cuff (NIBP100D, BIOPAC Systems, Inc., Goleta, CA, United States) and acquired at 500 Hz. Baroreflex sensitivity was calculated using the sequence method (Parati et al., 1988) by (1) extracting systolic blood pressures (SBP) and inter-beat intervals (IBI) from the BP trace and (2) identifying at least three consecutive beats in which an increase (or decrease) of at least 1 mmHg in SBP is followed by an increase (or decrease) of at least 5 ms in IBI. For each of these SBP-IBI sequences, the slope of the regression line was calculated when correlation coefficient was ≥0.85. BRS was the average of all slopes for each phase.

Heart rate and R-R intervals were recorded using a heart rate monitor (Polar RS800CX, FI-90440 Kempele, Finland). Ectopic beats in the R-R series were compensated for by interpolation of means to obtain normal-to-normal intervals. Mean heart rate and root mean square of the successive differences (RMSSD) were computed from the normal-to-normal intervals and spectral power in the high-frequency band (HF, 0.15–0.40 Hz) was computed using the Welch method after resampling normal-to-normal intervals at 4 Hz. All signal processing was performed using MATLAB^®^ 2015a (MathWorks, 160 Natick, MA, United States). Middle cerebral artery velocity was measured in the left middle cerebral artery trough left temporal window using a transcranial doppler (Spencer technology, Redmond, WA 98052-2559 United States) and acquired at 500 Hz. Finger arterial oxygen saturation was monitored using a finger oximeter (Wristox 3150 with 8000SM-WO Sensor, Nonin, Plymouth, MN, United States) and acquired at 0.5 Hz. Cerebral and muscular oxygenation was measured using near-infrared spectroscopy technology (NIRO-200-NX, Hamamatsu Photonics, Japan) and acquired at 1 Hz. Two detection and emission probes were located on the forehead and on the vastus lateralis in order to measure tissue oxygenation index (TOI), defined as the ratio of oxy-hemoglobin to change of total hemoglobin concentration, and relative concentration of total hemoglobin (tHb, normalized tissue hemoglobin index) in cerebral and muscular areas.

## Results

**FIGURE 1:**
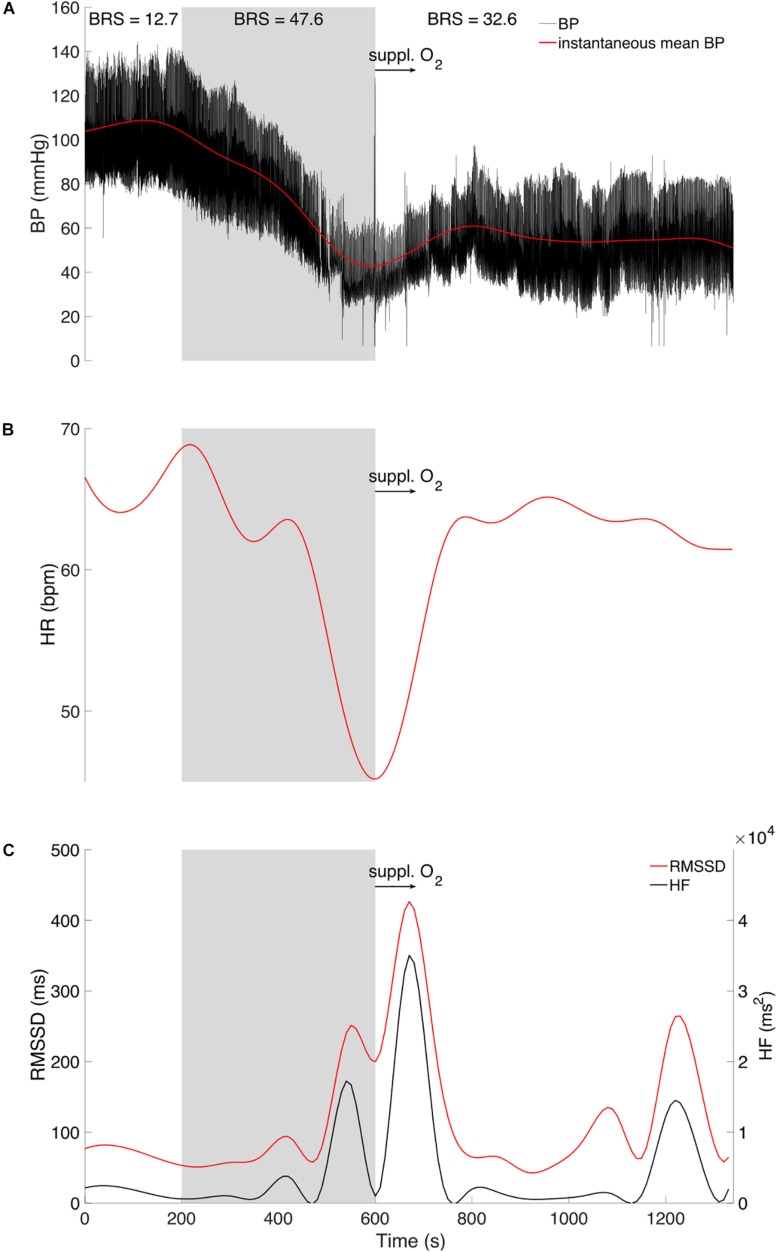
Original tracing of instantaneous **(A)** arterial blood pressure (BP, black trace) and mean value (red trace), **(B)** Heart rate (HR) in red tracing, and **(C)** Root mean square of the successive differences (RMSSD, red trace) and high frequency power (HF, black trace). Displayed for three phases: Baseline (0–200 s), vasovagal reaction (gray area, 200–600 s), and recovery period (600–1200 s) with brief 100% medical oxygen administration (black arrow, 600–700 s). Baroreflex sensitivity (BRS) is presented for each of the three phases.

**FIGURE 2:**
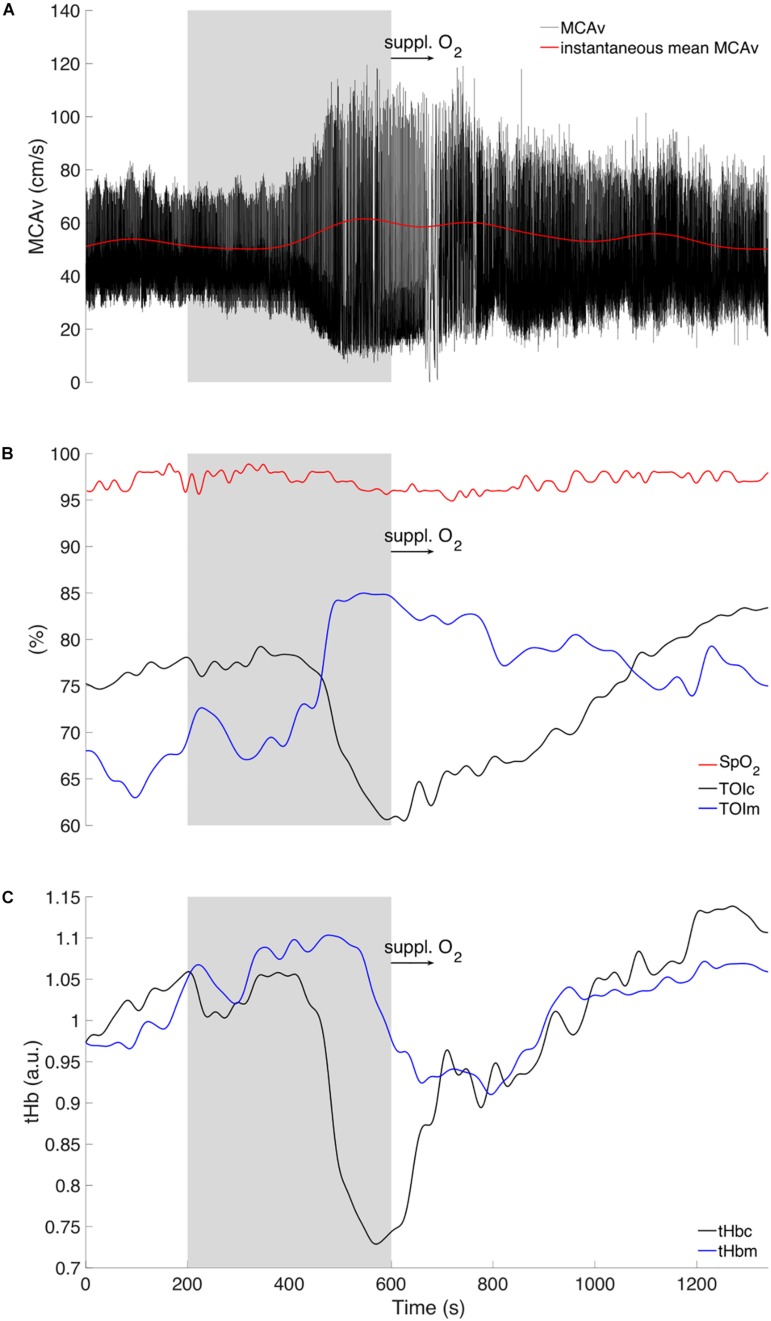
Original tracing of instantaneous **(A)** middle cerebral artery velocity (MCAv, black trace) and mean value (red trace). **(B)** Pulse oxygen saturation (SpO_2_) (red trace). Hemodynamic responses of cerebral tissue (TOI_c_) and muscular (TOI_m_) oxygenation index in black and blue tracings, respectively. **(C)** Tracings represent cerebral (tHb_c_, black trace) and muscular (tHb_m_, blue trace) total hemoglobin relative concentrations. Displayed for three phases: Baseline (0–200 s), vasovagal reaction (gray area, 200–600 s), and recovery period (600–1200 s) with brief 100% medical oxygen administration (black arrow, 600–700 s).

**FIGURE 3:**
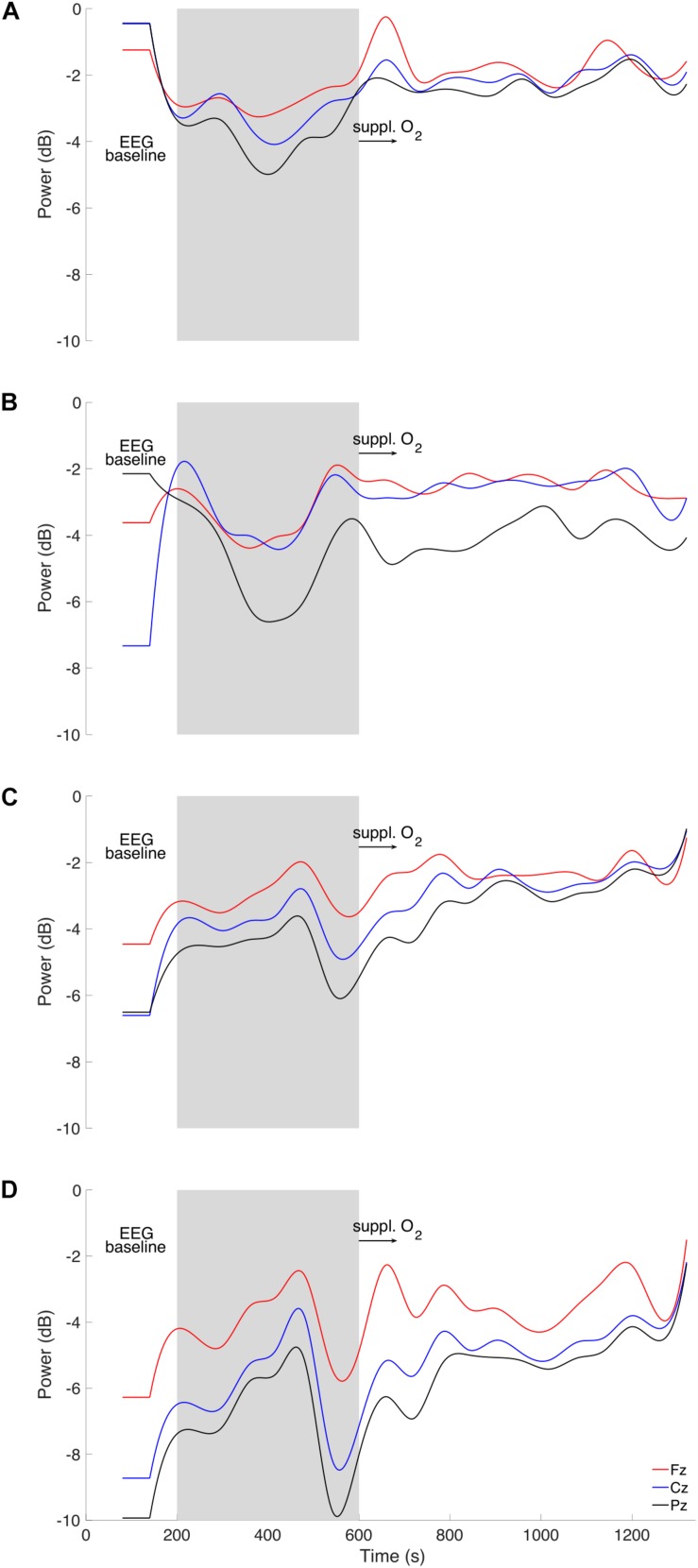
Time course of EEG **(A)** theta, **(B)** alpha, **(C)** beta, and **(D)** gamma power (in decibel; dB) for frontal (Fz, red), central (Cz, blue) and posterior (Pz, black) electrodes are displayed for three phases: Baseline (0–200 s), vasovagal reaction (gray area, 200–600 s), and recovery period (600–1200 s) with brief 100% medical oxygen administration (black arrow, 600–700 s).

The participant remained seated for 45 min during device installation and experimental trial preparation. He demonstrated a normal health state at baseline (0–200 s in Figures 1–3). The first physiological changes were observed at about 200 s with a progressive decrease in blood pressure, a drop in alpha power as well as a peak in HR and vastus lateralis TOI. The participant started having postural tone alterations when the vasovagal reaction occurred, but he did not lose consciousness. He communicated discomfort, felt dizzy and nauseous and looked pale. His vision darkened with flashing in his eyes and his hearing and concentration were altered. His symptoms resolved slowly during the recovery period (600–1200 s), after brief 100% medical oxygen administration (600–700 s).

**FIGURE 4:**
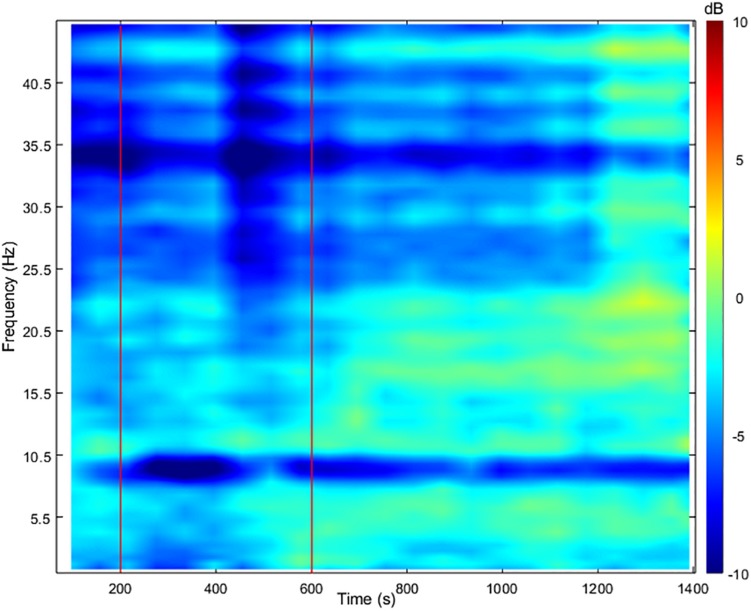
Change in time-frequency power spectrum from baseline on the posterior (Pz) electrode. Two vertical red lines delimit the vasovagal reaction (200–600 s).

His blood pressure started to progressively decrease at around 200 s (Figure 1A) with a simultaneous peak in HR (i.e., +5 bpm, Figure 1B), vastus lateralis TOI (i.e., +10%, Figure 2B), and systematic variations in EEG (i.e., drop in alpha power, Figures 3B, 4). After 200 s, the blood pressure showed classically reported behavior, with a progressive decrease (≈5 min), followed by steady low values (mean blood pressure ≈40 mmHg). Baroreflex sensitivity increased significantly during the vasovagal reaction. Bradycardia occurred simultaneously with the reduction in blood pressure. RMSSD and HF signals increased during, and shortly after the vasovagal reaction (Figure 1C). While peak systolic MCAv increased slightly, diastolic MCAv drastically decreased (<30 mmHg; Figure 2A), resulting in constant MCAv. Pulse oxygen saturation remained unchanged (Figure 2B). The cerebral oxygenation index decreased by ≈15% and total hemoglobin relative concentration by ≈25% (Figures 2B,C). However, the muscular oxygenation index increased by 20% in the vastus lateralis. Total hemoglobin in the frontal area decreased by 25%, while it remained stable in the vastus lateralis. EEG beta and gamma power decreased between 500 and 550 s, which corresponds to EEG flattening during cerebral hypoperfusion (Dijk et al., 2014; Figures 3C,D). EEG alpha power substantially decreased before the symptomatic vasovagal reaction (Figure 3B) in the frontal, central and posterior brain regions. This EEG switch from high to low frequencies corresponds to simultaneous EEG slowing (Heyer et al., 2016) and was associated with hypotension during pre-syncope. The EEG signal did not show any signs of epilepsy.

During the recovery period after brief 100% O_2_ inhalation (600–700 s), the participant remained unwell (with pallor and gastrointestinal discomfort). His blood pressure gradually increased but to values lower than baseline (Figure 1), whilst his heart rate increased nearly back to normal RMSSD and HF decreased back to baseline values, despite a small peak ≈10 min after vasovagal reaction. The diastolic MCAv increased progressively back to baseline values, whilst the systolic MCAv remained higher than baseline (Figure 2). The cerebral TOI and total relative hemoglobin concentration increased gradually to baseline. EEG alpha power returned to normal in the frontal and central regions following the vasovagal reaction but remained low in the posterior regions (Figure 3). The recovery period was associated with EEG slowing with an increase only in power of theta frequency (Figure 3A). EEG beta and gamma power returned to baseline, corresponding to the end of the “flat” phase. The participant remained conscious at all times.

## Discussion

This case report presents continuous and synchronous hemodynamic and EEG changes during a spontaneous and unexpected vasovagal reaction. Cerebral hypoperfusion, de-oxygenation and EEG alpha power in the fronto-central and posterior brain regions were decreased. The vasovagal reaction was associated with EEG flattening (i.e., reduced power in beta and gamma activity) followed by a “slow” phase (i.e., increased power in theta activity). These continuous recordings give insight to the succession of events before, during and following vasovagal reaction without syncope.

The peaks in HR and TOI in the vastus lateralis, with associated blood pressure decrease and EEG systematic variations, suggest that the onset of the vasovagal reaction was at around 200 s after the start of the recordings. The decrease in blood pressure and HR are likely due to the parasympathetic over-activation (RMSSD and HF increase) known to trigger vasovagal reactions, which in turn decrease cardiac output (Jørgensen et al., 1993). Baroreflex sensitivity increased drastically during vasovagal reaction, indicating that the auto-regulatory loop was overwhelmed by the large and sudden drop in blood pressure probably induced by a systemic vasodilation. The EEG amplitude decrease is not delayed when compared to blood pressure, which favors a connection between cortical areas and the medulla. If cerebral hypoperfusion had altered the EEG, its traces would have changed after the MCAv signal was detected, which in our case would have been around 400 s, whilst EEG traces dipped at around 200 s. Moreover, the electrocortical depression (expressed by a drop of theta and alpha power amplitudes) has previously been related to cerebral hypoperfusion and bradycardia (Dijk et al., 2014). Therefore, the posterior alpha power decrease may be interpreted as cerebral reactivity to visual and attentional dysfunction as reported by the participant. Cerebral hypoperfusion probably occurred primarily because of decreased blood pressure. The resulting cerebral hypoperfusion is considered as a primary driver in a vasovagal reaction. Overall, our data suggest that the onset of the vasovagal reaction at around 200 s was associated with a peak in heart rate and a progressive drop in blood pressure with a simultaneous drop in alpha power (i.e., desynchronization).

The participant reported being anxious in the reduced environment where the measurements took place. In addition, the participant’s equipment may have added to his anxiety. The start of the recordings may have triggered the vasovagal reaction and pre-syncope.

The EEG is among the first parameters to change and to recover at around 550 s, whilst blood pressure, heart rate, and MCAv remained abnormal. As a consequence, cardiac output and cerebral perfusion were reduced. In this case, cerebral hypoperfusion may be limited by the fact that the participant was seated (as opposed to standing) during the vasovagal reaction, which may explain why he did not lose consciousness. Despite limited orthostatic pressure, the parasympathetic over-activation (and sympathetic withdrawal in the muscles) still induced blood pooling in the lower limbs (Gunnar Wallin and Sundlöf, 1982), likely via systemic vasodilation (Lund et al., 2017). This can be seen from the NIRS traces, which showed marked decrease in the brain from 400 to 600 s and a light increase in the vastus lateralis over the same period. The data presented show the lasting effects of the vasovagal reaction with prolonged hypotension up to 6 min, during which SBP remained low (<80 mmHg). Patients with prolonged hypotension have longer EEG slowing phase during vasovagal reaction without syncope (Heyer, 2019). Moreover, EEG slowing was related to cerebral hypoperfusion and bradycardia (Dijk et al., 2014) and denotes changes of synaptic function due to cerebral ischemia during vasovagal syncope (Hofmeijer and van Putten, 2011). One may speculate that fast EEG switching to baseline values may explain why our participant did not lose consciousness. Our participant experienced vasovagal mechanisms with pre-syncope symptoms, but without progressing to syncope.

## Conclusion

We have presented simultaneous physiological recordings during spontaneous and unexpected vasovagal reaction without loss of consciousness in a young individual. The onset of the vasovagal reaction was associated with a peak in heart rate and progressive decrease in blood pressure, with a simultaneous drop in alpha power. Parasympathetic over-activation led to hypotension and cerebral hypoperfusion. His emotional status (anxiety and feeling of oppression) and concomitant prolonged hypotension and bradycardia with EEG flattening are in favor of a vasovagal mechanism without syncope. The recovery period was associated with EEG slowing and restoration of normal EEG pattern and arousal.

## Data Availability Statement

All datasets generated for this study are included in the article/supplementary material.

## Ethics Statement

The studies involving human participants were reviewed and approved by the Ethics Committee of Zurich, Switzerland (2018-00006). The patient/participant provided his written consent to participate in this study.

## Author Contributions

MA, NB, GM, and DB conceived and designed the research. MA performed the experiments and drafted the manuscript. MA, NB, HM, and JB analyzed the data and prepared the figures. MA, NB, JB, GM, and DB interpreted the results of the experiments. MA, NB, EN, JB, GM, and DB edited and revised the manuscript. All authors approved the final version of manuscript.

## Conflict of Interest

The authors declare that the research was conducted in the absence of any commercial or financial relationships that could be construed as a potential conflict of interest.
